# Supplementary material for: Remyelination alters the pattern of myelin in the cerebral cortex
Source: eLife. 2020 May 27;9:e56621. doi: 10.7554/eLife.56621 (PMC7292648; doi:10.7554/eLife.56621)
Supplement: Supplementary file 1. [file elife-56621-supp1.docx]

**Supplementary Table 1**: Summary of statistical tests and significance level for comparisons by comparison for each referenced figure panel.

| **Figure panel** | **Comparison** | **Statistical test** | **Significance level** |
| --- | --- | --- | --- |
| Figure 1J | Rate of oligodendrocyte loss  Cuprizone v. control  @ 3 weeks | N-way ANOVA with Bonferroni correction for multiple comparisons | *p* = 0.02014 |
|  | @ 4 weeks |  | *p* = 0.0000488 |
|  | @ 5 weeks |  | *p* = 0.0066 |
| Figure 1K | Rate of oligodendrocyte addition  Cuprizone v. control  @ 3 weeks | N-way ANOVA with Bonferroni correction for multiple comparisons | *p* = 0.0280 |
|  | @ 4 weeks |  | *p* = 0.0121 |
|  | @ 5 weeks |  | *p* = 0.000530 |
| Figure 2E | Rate of oligodendrocyte addition in control  0-100 μm v. 200-300 μm  @ 5 weeks | N-way ANOVA with Bonferroni correction for multiple comparisons | *p* = 0.0036 |
| Figure 2F | Rate of oligodendrocyte loss in cuprizone  0-100 μm v. 200-300 μm  @ 4 weeks | N-way ANOVA with Bonferroni correction for multiple comparisons | *p* = 0.0443 |
| Figure 2G | Rate of oligodendrocyte addition in cuprizone  0-100 μm v. 200-300 μm  @ 4 weeks | N-way ANOVA with Bonferroni correction for multiple comparisons | *p* = 0.0364 |
| Figure 3C | Displacement, Control cells,  All cells 0 - 100 μm v. Self-self 0 - 100 μm | N-way ANOVA with Bonferroni correction for multiple comparisons | *p* = 0.0137 |
|  | Displacement, Control cells,  All cells 0 - 300 μm v. Self-self 0 - 300 μm |  | *p* = 0.2837 |
|  | Displacement, Regenerated cells,  All cells 0 - 300 μm v. Self-self 0 - 300 μm |  | *p =* 8.95 x 10^-^*^7^* |
|  | Displacement, Regenerated cells,  All cells 0 - 100 μm v. Self-self 0 - 100 μm |  | *p =* 9.12 x 10^-7^ |
|  | Displacement, self-self 0-300 μm control v. regenerated |  | *p* = 1 |
|  | Displacement, all cells 0-300 μm control v. regenerated |  | *p* = 9.43 x 10^-5^ |
|  | Displacement, self-self 0-300 μm control v. regenerated |  | *p* = 1 |
|  | Displacement, all cells 0-100 μm control v. regenerated |  | *p* = 3.43 x 10^-5^ |
| Figure 3E | Number of myelin sheaths, remyelinating v. new control | Unpaired two-tailed t-test | *p* = 0.631 |
| Figure 3F | Total myelin length per cell, Remyelinating cell v. New control cell | Unpaired two-tailed t-test | *p* = 0.047 |
| Figure 3G | Individual myelin sheath length, Remyelinating cell v. New control cell | Unpaired two-tailed t-test | *p* = 0.014 |
| Figure 3I | Vector orientation, New control cells v. Remyelinating cells | Hodges-Ajne test of non-uniformity | *p* = 0.256 |
| Figure 3J | Average circular morphologies, New control cells v. Remyelinating cells | Kuiper two-sample test | *p* > 0.1, k = 462 |
| Figure 4B | *x-y* radius, New control vs. remyelinating cells territory | one-way ANOVA with Tukey’s HSD correction for multiple comparisons | *p* = 0.0415 |
|  | *x-y* radius, baseline vs. remyelinating cells territory |  | *p* = 0.0453 |
|  | *x-y* radius, baseline vs. new control |  | *p* = 0.9816 |
| Figure 4E | Proportion of overlap of regenerated oligodendrocyte territory with total baseline volume, Actual data v. randomized distribution of regenerated cell territories | One-way ANOVA | *p* = 0.0778 |
| Figure 4F | Proportion of novel territory encompassed by regenerated oligodendrocytes,  Actual data v. randomized distribution of regenerated cell territories | One-way ANOVA | *p* = 0.662 |
| Figure 6D | proportion of internodes, 0 v. >= 1 neighbor  @ baseline  control | N-way ANOVA with Bonferroni correction for multiple comparisons | *p* = 1.37 x 10^-6^ |
|  | proportions of internodes, baseline v. 8 weeks  control | N-way ANOVA | *p* = 1 |
| Figure 6E | proportions of internodes, stable v. novel  control | N-way ANOVA | *p* = 1 |
| Figure 6G | proportion of internodes, 0 v. >= 1 neighbor  @ baseline  cuprizone | N-way ANOVA with Bonferroni correction for multiple comparisons | *p* = 1.37 x 10^-6^ |
|  | proportions of internodes, baseline v. 8 weeks  cuprizone | N-way ANOVA | *p* = 1 |
| Figure 6E,G | proportions of internodes, control v. cuprizone  @ baseline | N-way ANOVA | *p* = 1 |
|  | proportions of internodes, control v. cuprizone  @ 8 weeks | N-way ANOVA | *p* = 1 |
| Figure 6H | proportion of internodes,  0 neighbors v. >= 1 neighbor  replaced | Unpaired two-tailed t-test with Bonferroni correction for multiple comparisons | *p* = 5.93 x 10^-7^ |
|  | proportion of internodes,  0 neighbors v. >= 1 neighbor  not replaced | Unpaired two-tailed t-test with Bonferroni correction for multiple comparisons | *p* = 1 |
|  | proportion of internodes,  0 neighbors v. >= 1 neighbor  not replaced | Unpaired two-tailed t-test with Bonferroni correction for multiple comparisons | *p* = 1 |
| Supplementary Figure 4F | OPC count ratios over time | Kruskall-Wallis one-way ANOVA | *p* = 0.0858 |
| Supplementary Figure 4G | Astrocyte count ratios over time | Kruskall-Wallis one-way ANOVA | *p* = 0.0056 |
|  | versus baseline:  cup1wk  cup2wk  cup3wk  rec1wk  rec2wk  rec3wk  rec5wk | Fisher’s least significant difference correction for multiple comparisons | *p* = 1  *p* = 0.573  *p* = 0.031  *p* =0.005  *p* = 0.005  *p* = 0.041  *p* = 0.034 |
| Supplementary Figure 5D | # of myelin sheaths undergoing retraction v. extension, control | Unpaired two-tailed t-tests with Bonferroni correction for multiple comparisons | *p* = 4.11 x 10^-7^ |
|  | # of myelin sheaths undergoing retraction v. extension, remyelinating | Unpaired two-tailed t-tests with Bonferroni correction for multiple comparisons | *p* = 0.00119 |
|  | Net length change, extensions v. retractions, control | Unpaired two-tailed t-tests with Bonferroni correction for multiple comparisons | *p* = 0.175 |
|  | Net length change, extensions v. retractions, remyelinating | Unpaired two-tailed t-tests with Bonferroni correction for multiple comparisons | *p* = 0.407 |
|  | Net length change in extensions, control v. remyelinating | Unpaired two-tailed t-tests with Bonferroni correction for multiple comparisons | *p* = 0.444 |
|  | Net length change in retractions, control v. remyelinating | Unpaired two-tailed t-tests with Bonferroni correction for multiple comparisons | *p* = 1.16 |
| Supplementary Figure 5E | # of lost sheaths, control v. remyelinating cells | Unpaired two-tailed t-test | *p* = 0.907 |
| Supplementary Figure 5F | Reduction in process length, control v. remyelinating cells | Unpaired two-tailed t-test | *p* = 0.474 |
| Supplementary Figure 5I | Absolute value of net total sheath length change, 0-4 days v. 8-14 days | N-way ANOVA with Bonferroni correction for multiple comparisons | *p* = 2.28 x 10^-6^ |

**Supplementary Table 1**: Summary of statistical tests and significance level for comparisons by comparison for each referenced figure panel.
